# The increased sensitivity of CTLs induced by vaccinia vector is developed as intrinsic feature in vivo and independent of microenvironment in vitro

**DOI:** 10.1186/1742-4690-9-S2-P252

**Published:** 2012-09-13

**Authors:** Z Hu, C Qiu, Y Wan, J Xu

**Affiliations:** 1 Shanghai Public Health Clinical Center, Fudan University, Shanghai, China; 2 Institutes of Biomedical Sciences, Fudan University, Shanghai, China

## Background

DNA prime-vaccinia viral vector (rVV) boost has been widely used as an effective regimen for T cell vaccine. This regimen could efficiently enhance the potency of immunogen-specific T cell responses with minimizing responses to vectors. In addition, we and others found that the sensitivity for T cells responding to antigenic stimulation is elevated by this vaccination regimen. However, it remains unknown whether the increased sensitivity of CD8+ T cells is gained during the activation and differentiation as intrinsic property or is resulted from the influence of microenvironment.

## Methods

The magnitude, potency and sensitivity of T cell responses induced by different vaccine regimens were compared by using ELISpot and ICS. Purified CD8+ T cells from each vaccination regimen were mixed with splenocytes from naïve or mock rVV immunized mouse and determined for their sensitivities. Statistical analysis was performed by using Prism5.0 software.

## Results

As expected, the magnitude, potency and sensitivity of T cell responses induced by DNA prime-rVV boost is 20-, 15- and 100- fold higher than that in group with repeated DNA boost, respectively. Interestingly, the sensitivity of CD8+ T cells purified from each vaccination regimen group is comparable before and after the mixture with splenocytes from either naïve or mock rVV immunized mouse, suggesting that the removal of inflammatory microenvironment did not decrease the sensitivity of CD8+ T cell responses. Accordingly, the addition of vaccinia induced microenvironment to CD8+ T cells purified from DNA group does not enhance their sensitivity.

## Conclusion

Our results demonstrated that the increased sensitivity of CD8+ T cells by DNA prime-rVV boost regimen is intrinsic property and likely to be developed and gained during the activation and differentiation in vivo by vaccine, this effect is independent with inflammatory microenvironment in memory status. These findings have important implications for the design of new vaccine and immunization strategies.

